# Effectiveness of biological therapy in severe asthma: a retrospective real-world study

**DOI:** 10.3325/cmj.2025.66.3

**Published:** 2025-02

**Authors:** Marina Lampalo, Anamarija Štajduhar, Dina Rnjak, Nikola Ferara, Hana Safić Stanić, Sanja Popović-Grle

**Affiliations:** 1 Clinical Department for Lung Diseases Jordanovac, Zagreb University Hospital Center, Zagreb, Croatia; 2 Department of Pulmonology, Zagreb University Hospital Center, Zagreb, Croatia; 3 University of Zagreb School of Medicine, Zagreb, Croatia; 4 Croatian Institute for Transfusion Medicine, Zagreb, Croatia

## Abstract

**Aim:**

To compare the effectiveness of different biologic medications for the treatment of severe asthma.

**Methods:**

We retrospectively collected data on 74 patients treated with one of four different biologics (omalizumab, mepolizumab, reslizumab, or benralizumab) at the Jordanovac Clinic for Pulmonary Diseases, Zagreb, Croatia for at least two years. The patients were compared in terms of the number of exacerbations, dose of oral corticosteroids (OCS), asthma control test (ACT), forced expiratory volume in 1 second (FEV_1_), forced vital capacity, fraction of exhaled nitric oxide (FeNO), number of blood eosinophils, and total immunoglobulin E (IgE).

**Results:**

All treatment outcome measures, except FeNO, significantly improved in the two-year period (*P* < 0.001). The number of acute exacerbations decreased in all groups. Reslizumab-treated patients showed the greatest improvement in ACT score, and the omalizumab-treated patients experienced the least (*P* = 0.018). The benralizumab group showed the greatest reduction in eosinophil number, and the omalizumab group the lowest (*P* < 0.001). The mepolizumab group showed the greatest improvement in FEV_1_.

**Conclusion:**

Both anti-IgE and anti-IL-5 treatments effectively reduced exacerbation rates, OCS daily needs, and symptom burden. Multiple predictive biomarkers are needed for the best individual monitoring.

In severe asthma patients, the disease remains uncontrolled despite optimized medical therapy and treatment of attributable factors. Uncontrolled asthma represents an important medical, physical, emotional, and societal burden.

The severe asthma phenotype is determined based on blood eosinophils, immunoglobulin E (IgE), allergy tests, and the fraction of exhaled nitric oxide (FeNO). Depending on the presence or absence of these markers, severe asthma can be divided into type 2 (T2) and non-type 2, respectively (1). Specific biological medications have been developed primarily for T2 severe asthma phenotype (eg, early-onset allergic and late-onset eosinophilic asthma): anti-interleukin 5 (IL5) (mepolizumab, reslizumab), anti-IL5 receptor (anti-IL5R) (benralizumab), anti-IL4 (dupilumab), and anti-IgE therapy (omalizumab) (1). Biological therapy has significantly improved severe asthma treatment, but there are still many knowledge gaps, especially concerning the guidelines for the treatment of patients with overlapping biomarkers, eg, elevated eosinophils and IgE (1). Furthermore, it remains unknown what the differences are between anti-eosinophilic treatments, what advantages some biologics have over the others, or in which cases one of them stands out as a therapy of choice. Although there are many studies about the efficacy of individual biologics, there is a lack of direct comparison studies, and the choice of therapy is based on current guidelines and professional opinion and experience (1).

Given the unlikely prospect of future head-to-head comparison studies, our study aimed to retrospectively analyze data from our severe asthma patients treated with various biologics (omalizumab, mepolizumab, reslizumab, and benralizumab) for a minimum of two years.

## Methods

### Study design

Our study enrolled 74 patients treated with one of the four different biologics for severe uncontrolled asthma at the Jordanovac Clinic for Pulmonary Diseases, Zagreb, during at least two years from 2010 to 2023. The indication for treatment was established by the treating physician and approved by the Multidisciplinary Team for Severe Asthma at the Clinic. The study was approved by the Medical Ethics Committee of University Hospital Center Zagreb. Each participant signed an informed consent.

Sixteen patients (21.6%) were treated with benralizumab, 22 (29.7%) with mepolizumab, 20 (27.0%) with reslizumab, and 16 (21.6%) with omalizumab according to the criteria of the Croatian Health Insurance Fund. We collected the data on baseline demographics, lung function measurements, eosinophil number, total IgE levels, comorbidities, and standard asthma treatment according to the introduced biologic treatment (Table 1). In every patient, data were obtained on the number of acute exacerbations, asthma control test (ACT) results, the dose of oral corticosteroids (OCS) used for the management of asthma, lung function spirometry parameters – forced expiratory volume in 1 second (FEV_1_) and forced vital capacity (FVC), fractional exhaled nitric oxide (FeNO), and total IgE in serum at three, six, nine, 11, and 24 months from the initiation of treatment. General information about the participants was obtained from the patients` medical records or during an interview.

**Table 1:** Baseline demographics, lung function measurements, eosinophil number, total IgE levels, comorbidities and standard asthma treatment according to introduced biologic treatment*

**Variable**	**Benralizumab (n = 16)**	**Mepolizumab (n = 22)**	**Reslizumab** **(n = 20)**	**Omalizumab (n = 16)**	**P**
Age in years, median (range)	60.5 (48.5-66.5)	57.0 (41.0-67.0)	58.0 (50.0-69.0)	55.0 (41.5-69.0)	0.902^§^
Women (%)	7	17	11	9	0.077^†^
Smoking habit, n					0.342^†^
non-smoker	7	13	10	12	
past smoker	9	7	6	8	
active smoker	0	2	0	0	
Body mass index, mean (standard deviation)	27.74 (4.35)	26.32 (4.70)	25.93 (3.96)	26.78 (5.26)	0.705^‡^
Comorbidities, n					
Hypertension	6	9	6	5	0.728^†^
CRS	5	7	8	0	0.007^†^
NP	8	8	9	4	0.116^†^
AD	0	0	1	3	0.122^†^
ARC	0	5	1	7	0.024^†^
Anxiety/depression	1	3	2	0	0.368^†^
Diabetes	3	4	3	3	0.988^†^
GERD	4	2	3	1	0.285^†^
Osteoporosis	3	5	5	2	0.457^†^
Heart failure	0	0	1	0	0.299^†^
EGPA	1	2	2	0	0.478^†^
Obesity	0	3	0	0	0.060^†^
Treatment, n					
ICS/LABA	1	3	1	1	0.727^†^
ICS/LABA/LTRA	0	3	1	1	0.405^†^
ICS/LABA/LAMA	3	10	6	4	0.194^†^
ICS/LABA/LAMA/LTRA	12	6	7	13	0.014^†^
SABA	0	4	3	2	0.296^†^
Theophylline	6	4	6	7	0.481^†^
OCS	16	20	14	19	0.515^†^
OCS (dose; median)	6 (4-16)	4 (2-8)	4 (2.5-4.75)	4 (2-8)	0.485^c^
Asthma control, mean (standard deviation)	12.0 (5.5)	12.6 (5.4)	14.8 (4.3)	14.7 (4.6)	0.263^‡^
Acute exacerbations in previous year, median (range)	3.5 (1.0-4.0)	4.0 (3.0-6.0)	4.0 (2.3-5.0)	1.0 (1.0-2.8)	<0.001^§^
VC (% predicted), mean (standard deviation)	77.4 (15.0)	80.0 (19.0)	76.8 (16.9)	77.9 (17.1)	0.943^‡^
FEV_1_ (% predicted), median (range)	45.0 (40.3-56.8)	51.3 (47.0-61.0)	53.5 (44.0-61.0)	54.9 (41.2-65.2)	0.556^§^
DLCO (% predicted), mean (standard deviation)	88.6 (19.2)	78.5 (16.4)	78.6 (16.0)	74.5 (22.7)	0.249^‡^
FeNO (ppb), median (range)	55.0 (40.5-131.3)	50.0 (27.0-96.5)	58.0 (41.5-69.6)	43.0 (15.0-59.0)	0.179^§^
Eo (μL^−1^), median (range)	570 (420-1030)	740 (660-1030)	680 (378-835)	340 (145-515)	<0.001^§^
Total IgE (kU/L), median (range)				322 (230-661)	na
PaO_2_ (mmHg), median (range)	81.5 (71.0-86.5)	79.0 (67.0-82.0)	75.4 (65.7-78.8)	78.0 (70.3-86.5)	0.183^§^
Hgb (g/L), mean (standard deviation)	144 (17)	142 (12)	139 (14)	140 (18)	0.836^‡^
MCV (fL), mean (standard deviation)	87 (3.5)	88 (3.9)	88 (3.4)	87 (5.8)	0.914^‡^
RDW (%), median (range)	13.9 (13.5-14.3)	13.3 (13.0-14.0)	13.6 (13.2-14.0)	13.0 (12.9-14.0)	0.250^§^

*Abbreviations: CRS – chronic rhinosinusitis; NP – nasal polyposis; AD – atopic dermatitis; ARC – rhinoconjunctivitis; GERD – gastroesophageal reflux disease; EGPA – eosinophilic granulomatosis with polyangiitis; ICS – inhaled corticosteroids; LABA – long-acting beta agonist; LTRA – leukotriene receptor antagonists; LAMA – long-acting muscarinic antagonists; SABA – short-acting beta agonists; OCS – oral corticosteroids dose; VC – vital capacity; FEV_1_ – forced expiratory volume in 1 second; DLCO – diffusion capacity for carbon monoxide; FeNO – fraction of exhaled nitric oxide; Eo – eosinophils; IgE – immunoglobulin E; Hgb – hemoglobin; MCV – mean corpuscular volume; RDW – red cell distribution width; PaO_2_ – the oxygen pressure in arterial blood.

†χ^2^ test.

‡ANOVA.

§Kruskal-Wallis ANOVA.

### Statistical analysis

The normality of distribution was tested with a Kolmogorov-Smirnov test. Categorical variables are presented as counts and proportions, and continuous variables as means with standard deviations (SD) or medians with interquartile ranges (IQR). Differences between the groups in categorical data were assessed with a χ^2^ test, and those in continuous data with an analysis of variance (ANOVA) or Kruskal-Wallis ANOVA with *post-hoc* tests. Repeated-measures ANOVA was used to test for differences in time dynamics of outcome measures between the groups. The Kaplan-Meier survival analysis was used to test the difference in time-to-first event between the groups (time-to-first exacerbation, time-to-first reduction in OCS). *P* < 0.05 was considered statistically significant, corrected for multiple comparisons. The analysis was performed with STATISTICA version 12 (StatSoft, Inc., Tulsa, OK, USA) and MedCalc® Statistical Software, version 20.015 (MedCalc Software Ltd, Ostend, Belgium).

## Results

The treatment groups were comparable in terms of the most of the baseline characteristics (Table 1). All participants were adults, with a slight female predominance (59%), and the mean age was 55 years. Significant differences were found in the proportions of chronic rhinosinusitis (CRS) (*P* = 0.007), which was not present in the omalizumab group, and allergic rhinitis (AR), which was not diagnosed in the benralizumab group (*P* = 0.024). Before the start of the treatment, patients treated with benralizumab and omalizumab received significantly higher proportions of inhaled corticosteroids/long-acting beta agonist/long-acting muscarinic antagonists/leukotriene receptor antagonists (ICS/LABA/LAMA/LTRA) combination than those treated with mepolizumab and reslizumab (*P* = 0.014). Other treatment options were similarly distributed between the groups (*P* > 0.10 for all). All groups received a comparable dose of OCS, with a median dose ranging between 4 and 6 mg/day (*P* = 0.485). The omalizumab group had the fewest acute exacerbations (AE) during the year preceding the start of treatment (median 1.0, *P* < 0.05 compared with other groups). The benralizumab group had significantly fewer AE than the mepolizumab group (median 3.5 and 4.0, *P* < 0.05). The mepolizumab and reslizumab groups had the most AE (median 4.0 for both). As expected, there was also a significant difference in the number of blood eosinophils between the groups (*P* < 0.001), with the lowest number in the omalizumab group (median 340/μL) and highest in the mepolizumab group (median 740/μL). No significant difference was observed between the three anti-IL-5 treatment groups (*P* > 0.05 for all intergroup comparisons).

**Figure 1:**
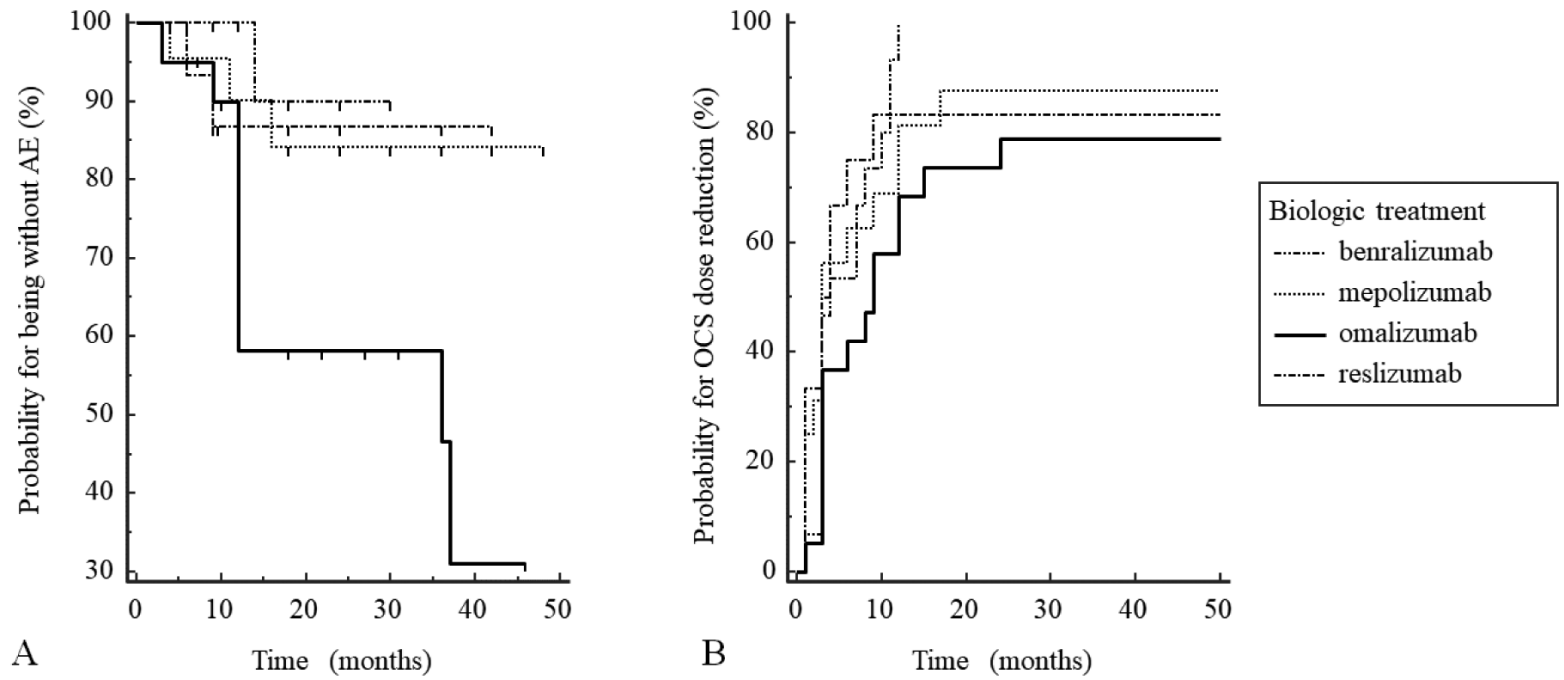
Kaplan-Meier survival analysis for the time-to-first acute exacerbation (AE) (**A**) and time-to-first oral corticosteroids (OCS) reduction (**B**) according to biologic treatment group.

During the two-year treatment period, all treatment outcome measures, except FeNO, significantly improved (*P* < 0.001) (Table 2, Figure 1B). The number of AE significantly dropped in all groups, reaching 0/year in all anti-IL-5 groups and 0.5/year in the omalizumab group (*P* < 0.001). The positive trend continued in all anti-IL-5 groups even after 14 months of treatment.

**Table 2:** Outcome measures in different biologic treatment groups during the two-year period

**Outcome measure**	**Benralizumab (n = 16)**	**Mepolizumab (n = 22)**	**Reslizumab** **(n = 20)**	**Omalizumab (n = 16)**	**P**
ACT, mean (standard deviation)					
baseline	12.0 (5.5)	12.6 (5.4)	14.8 (4.3)	14.7 (4.6)	
3 months	20.8 (3.7)	18.4 (4.5)	20.7 (5.3)	18.4 (5.4)	<0.001^†^
6 months	21.1 (5.2)	20.7 (3.7)	21.9 (4.3)	19.5 (3.8)	0.018^‡^
9 months	22.9 (4.0)	21.2 (3.4)	23.5 (3.0)	19.9 (4.4)	
12 months	22.1 (3.4)	22.1 (2.9)	24.3 (1.7)	20.0 (4.4)	
24 months	22.5 (3.3)	22.4 (2.2)	24.2 (1.6)	21.2 (4.6)	
Eo/microliter, median (range)					
baseline	570 (420-1030)	740 (660-1030)	680 (378-835)	340 (145-515)	<0.001^†^
after treatment	0 (0-0)	80 (50-120)	30 (23-40)	150 (103-300)	<0.001^‡^
FEV_1_ (% predicted), mean (standard deviation)					
baseline	51.6 (19.4)	53.1 (16.3)	53.5 (10.9)	55.4 (17.3)	
3 months	72.9 (19.1)	60.5 (18.1)	70.2 (14.1)	55.9 (16.1)	<0.001^†^
6 months	70.6 (22.6)	74.4 (27.8)	70.9 (16.9)	65.4 (18.3)	0.001^‡^
9 months	72.1 (22.2)	76.9 (25.7)	70.8 (13.6)	63.8 (19.5)	
12 months	71.5 (23.9)	77.0 (23.6)	75.6 (15.8)	64.4 (18.4)	
24 months	64.3 (18.6)	77.0 (28.7)	73.8 (14.9)	56.3 (20.6)	
OCS (dose), median (range)					
baseline	6 (4-16)	4 (2-8)	4 (2.5-4.75)	4 (2-8)	
3 months	4 (2-7)	0 (0-4.5)	4 (0-4)	4 (1-8)	<0.001^†^
6 months	2 (0-7)	0 (0-4)	0 (0-2)	2 (0-4)	0.081^‡^
9 months	1 (0-12)	0 (0-4)	0 (0-2)	2 (0-4)	
12 months	0 (0-2)	0 (0-3)	0 (0-0.5)	2 (0-4)	
24 months	0 (0-3.5)	0 (0-2)	0 (0-0)	0 (0-2.35)	
VC (% predicted), mean (standard deviation)					
baseline	77.4 (15.0)	80.0 (19.0)	76.8 (16.9)	77.9 (17.1)	<0.001^†^
after treatment	88.3 (14.6)	97.0 (18.1)	85.4 (18.7)	78.7 (12.4)	0.023^‡^
FeNO (ppb), median (range)					
baseline	55.0 (40.5-131.3)	50.0 (27.0-96.5)	58.0 (41.5-69.6)	43.0 (15.0-59.0)	0.118^†^
after treatment	77.0 (28.5-107.8)	29.0 (17.0-41.5)	35.0 (25.0-80.5)	52.0 (28.0-105.0)	0.025^‡^
Total IgE (kU/L), median (range)					na
baseline				322 (230-661)	
after treatment				287 (173-475)	
PaO_2_ (mmHg), mean (standard deviation)					
baseline	79.2 (9.6)	75.4 (10.4)	71.2 (11.7)	77.7 (11.5)	<0.001^†^
after treatment	82.3 (7.4)	82.1 (6.0)	80.3 (9.6)	99.5 (18.8)	0.002^‡^
Acute exacerbations, median (range)					
baseline	3.5 (1.0-4.0)	4.0 (3.0-6.0)	4.0 (2.3-5.0)	1.0 (1.0-2.8)	<0.001^†^
after treatment	0.0 (0.0-0.0)	0.0 (0.0-0.0)	0.0 (0.0-0.0)	0.5 (0.0-1.0)	<0.001^‡^
DLCO, mean (standard deviation)					
baseline	88.6 (19.2)	78.5 (16.4)	78.6 (16.0)	74.5 (22.7)	<0.001*
after treatment	94.0 (18.4)	88.9 (18.7)	86.4 (15.1)	85.1 (28.1)	0.714^‡^

*Abbreviations: ACT – asthma control test; Eo – eosinophils; FEV_1_ – forced expiratory volume in 1 second; OCS – oral corticosteroids dose; VC – vital capacity; FeNO – fraction of exhaled nitric oxide; IgE – immunoglobulin E; PaO_2_ – the oxygen pressure in arterial blood; DLCO – diffusion capacity for carbon monoxide.

*repeated measures ANOVA, time change.

‡repeated measures ANOVA, interaction of time*group.

**Figure 2:**
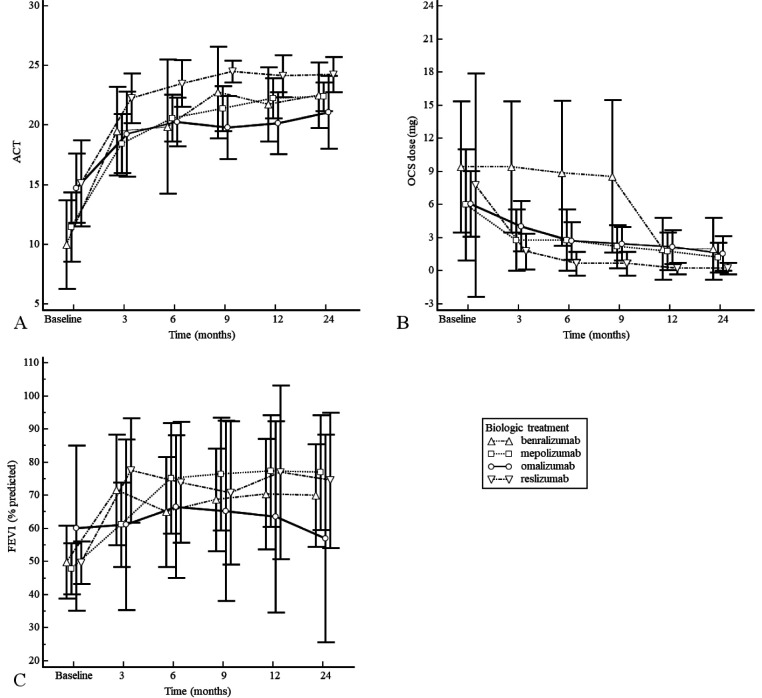
Change in outcome measures during the treatment with biologics: (**A**) asthma control test (ACT), (**B**) oral corticosteroids dose (OCS), and (**C**) forced expiratory volume in 1 second (FEV_1_).

**Figure 3:**
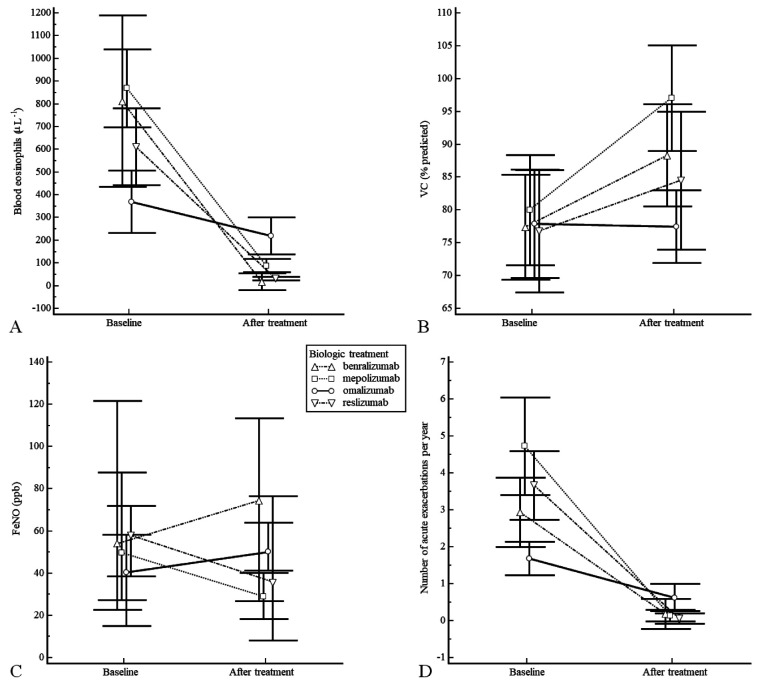
Change in outcome measured after the treatment with biologics; (**A**) number of blood eosinophils, (**B**) forced vital capacity (FVC), (**C**) fraction of exhaled nitric oxide (FeNO), (**D**) number of acute exacerbations.

There were no new AE in any of the anti-IL-5 treated groups after 14 months of treatment (Figure 1A). Survival analysis showed a significant difference in time-to-first exacerbation between biologic treatments (*P* = 0.028, Kaplan-Meier survival analysis; Figure 1A), with a significantly higher hazard ratio (HR) in patients on omalizumab compared with benralizumab (HR = 6.04, 95% CI 1.38-26.47) and mepolizumab (HR = 3.72, 95% CI 1.07-12.98). There were no other significant differences between the groups. There was no difference in time-to-first OCS reduction between biologics (*P* = 0.270, Kaplan-Meier survival analysis; Figure 1B). The greatest improvement in ACT score was observed in the reslizumab group and the least in the omalizumab group (*P* = 0.018; Figure 2A). The greatest reduction in eosinophil number was observed in the benralizumab group and the lowest in the omalizumab group (*P* < 0.001; Figure 3A). The greatest improvement in FEV_1_ was noted in the mepolizumab group, and a marginal improvement was noted in the omalizumab group (*P* = 0.001; Figure 2C). Similarly, the best improvement in FVC was observed in the mepolizumab group, while no improvement was observed in the omalizumab group (*P* = 0.023; Figure 3B). FeNO showed two different patterns: an increasing trend in the benralizumab and omalizumab groups and a decline in the mepolizumab and reslizumab groups (*P* = 0.025; Figure 3C).

## Discussion

Our study showed that both anti-IgE and anti-IL-5 treatments effectively reduced exacerbation rates, OCS daily needs, and symptom burden. A systematic review by Bourdin et al indirectly compared benralizumab and mepolizumab, revealing similar efficacy profiles of these two biologics concerning a reduction in exacerbation rates and an improvement in FEV_1_ (3). However, another review suggested that benralizumab may be less effective than reslizumab regarding the Asthma Quality of Life Questionnaire and Asthma Control Questionnaire scores, FEV_1_, and exacerbations (4). Mepolizumab significantly reduced the number of exacerbations compared with benralizumab and reslizumab (5). In other analyses, dupilumab was superior to other anti-eosinophilic biologics (6,7). Nonetheless, a meta-analysis encompassing randomized clinical trials of anti-IL5 treatments over the 1990-2015 period found no clear distinctions between these drugs (8). An indirect comparison by Cockle showed no difference between omalizumab and mepolizumab in the treatment of clinically significant exacerbations (9). Kotisali et al compared the treatment outcomes for anti-IL5 therapy and found a significant reduction in OCS use only in the reslizumab group. Still, all asthma biologics significantly reduced the total number of exacerbations (10). Furthermore, a meta-analysis concluded that anti-eosinophilic biologic therapy was superior to placebo in preventing asthma exacerbations but did not reveal significant differences between various drugs (11). Notably, there have been no direct head-to-head comparisons between biologics in the context of severe asthma, and there are limited studies comparing the effects of biologics in real-world settings.

In Croatia, five biologic agents have been available since 2024, but our study exclusively enrolled patients who had been consistently treated with anti-IgE (omalizumab) and anti-IL-5 biologic therapy (mepolizumab, benralizumab, reslizumab) for a minimum of two years.

Patients did not significantly differ in terms of baseline characteristics. Most of them were non-smokers, middle-aged, and had various comorbidities. Notably, a significantly higher proportion of patients with chronic and allergic rhinosinusitis received one of the anti-IL-5 drugs, compared with omalizumab. Previous studies indicated the effectiveness of all these biologics in treating nasal polyposis and eosinophilic upper airway inflammation in severe asthma patients irrespective of their atopic status (4-6). None of our patients with severe asthma and eosinophilic granulomatosis with polyangiitis was treated with omalizumab due to concerns about vasculitis exacerbation. However, vasculitis exacerbation can more likely be attributed to a reduction in steroid doses rather than to omalizumab (7,8). Only a minority of patients suffered from osteoporosis, obesity, or diabetes, despite long-term high-dose inhaled corticosteroid and OCS treatment, which reflects our proactive efforts in early recognition and treatment of these steroid-related complications (9,10).

Before the introduction of biologic therapy, all patients experienced multiple AE annually, had poor symptom control (as indicated by ACT scores below 20), moderately-to-severely impaired lung function, and high blood eosinophilia. Patients treated with omalizumab had elevated total IgE or positive specific IgE. Differences in treatment regimens between the groups, particularly related to antileukotrienes, can be attributed to their higher use in patients with atopic status, who were subsequently mostly treated with omalizumab, in line with the Croatian recommendations and health care insurance provider conditions. This reasoning, along with scientific rationale, underlies the fact that the highest baseline blood eosinophilia was present in participants on anti-IL-5 therapy.

The results of our study align with those of some earlier research (11-15). We observed significant improvements in all measured parameters, except FeNO, which remained similar in both omalizumab and anti-IL-5 groups, consistent with earlier studies (11-15). The most substantial progress was noted for the anti-IL-5 medications, particularly reslizumab. We also speculate that the considerable treatment benefit in obese patients receiving reslizumab may be attributed to the adjustment of the drug dose based on body mass index. In almost all anti-IL-5 patients, the number of yearly exacerbations was reduced to zero, and the OCS dose was significantly reduced after just three months, with most patients discontinuing OCS after one year. In contrast, omalizumab exhibited an inferior steroid-sparing effect but still led to a reduction in OCS dose to half of the baseline dose, consistent with some earlier trials (16,17). Eosinophil counts, one of the most readily measurable elements used to select eligible patients for biologic treatment, normalized in all anti-IL-5 patients, most notably in the benralizumab group, but remained marginally elevated in the omalizumab group, with high variability over time. Despite omalizumab’s reducing total IgE levels, elevated blood eosinophils could lead to a slower steroid dose reduction and slightly worse symptom control, as this asthma type-2 inflammatory pathway remains active. In these patients, a switch to another biologic therapy has proven beneficial in our ongoing study. Lung function, assessed through FEV_1_ and FVC spirometry parameters, improved most in the anti-IL-5 group, especially in patients treated with mepolizumab compared with omalizumab. This is in line with the results from the INNOVATE, MENSA, and CALIMA studies, and the trial by Castro et al (18-21). The most substantial increase in FEV_1_ was observed three months after the initiation of biologic treatment and was maintained subsequently.

The FeNO levels did not improve in the mepolizumab and reslizumab groups. Nevertheless, given the clinical improvement in all the studied groups, irrespective of FeNO levels, we conclude that FeNO is a useful marker of type-2 immunologic response and a valuable tool for selecting patients for dupilumab treatment. However, it likely has a limited impact on monitoring the treatment response to anti-IgE and anti-IL-5 therapies in severe asthma patients. While ACT scores, lung function values, and blood eosinophils evidently improved shortly after the initiation of biologic treatment, a reduction in OCS was more gradual. Therefore, we believe it reasonable to assess the response to biologics in asthma patients no earlier than 3-6 months after the initiation of biologic therapy. Also, we did not record any major side effects of anti-IgE and anti-IL-5 therapy during the two-year period.

Several limitations of our study should be acknowledged. This is a retrospective single-center study assessing the outcomes of a relatively small group of patients in a relatively short period of two years. Consequently, definitive conclusions about the impact of anti-IgE and anti-IL-5 therapy in severe asthma, especially concerning disease complications, life expectancy, or mortality, cannot be drawn. Furthermore, 8 patients who discontinued omalizumab or some anti-IL-5 treatment after one year due to ineffectiveness were not included in our statistical analysis. Nevertheless, our study results are consistent with those of other large clinical trials, and we evaluated multiple relevant indicators for severe asthma. As noted by Numata et al (22), the variation in the efficacy of omalizumab and anti-IL-5 therapy likely stems from selection bias. In Croatia, as in other countries, omalizumab was the first biologic to enter the market, which is why many patients who would have been better suited for anti-IL-5 biologics initially received omalizumab (22).

In conclusion, both anti-IgE and anti-IL-5 treatments are highly effective in reducing exacerbations, symptom burden, and daily OCS requirements in severe asthma patients. At the moment, we are still unable to provide the definitive answer as to which biologic is a better choice in general in the T2 asthma group, and we still lack precise guidelines for the most suitable biologic therapy. Also, decisive factors in making this decision are often the cost of treatment, application route, or number of treatment visits needed. Accurate asthma phenotyping also remains a challenge. Moreover, there is a need for effective biologic therapies for patients with severe non-T2 asthma, a large group with unmet needs.
